# Prognostic value of EGFR and p-EGFR in nasopharyngeal carcinoma: A systematic review and meta-analysis

**DOI:** 10.1097/MD.0000000000028507

**Published:** 2022-01-21

**Authors:** Xiaohua Hong, Guangyao Wang, Guanglan Xu, Wei Shi, Tongbiao Wang, Zhen Rong, Chunmei Mo

**Affiliations:** a Guangxi University of Chinese medicine, NanNing Guangxi, China; b The First Affiliated Hospital of Guangxi University of Chinese Medicine, Nanning Guangxi, China; c Bao’an Authentic TCM Therapy Hospital, Shenzhen Guangdong, China.

**Keywords:** epidermal growth factor receptor, nasopharyngeal carcinoma, phosphorylated epidermal growth factor receptor, prognosis, systematic review and meta-analysis

## Abstract

**Purpose:**

To evaluate the prognostic effect and clinical significance of epidermal growth factor receptor and its phosphorlated form (EGFR/p-EGFR) in nasopharyngeal carcinoma.

**Methods:**

A systematic review and meta-analysis was designed. We visited PubMed, Embase, China National Knowledge Infrastructure Database, Database of Chinese sci-tech periodicals, WanFang Database, and China Biology Medicine disc to search for Chinese and English publications of prospective studies and retrospective studies investigating the association of EGFR/p-EGFR and nasopharyngeal carcinoma prognosis from inception to April 2021. The inclusion criteria were that the samples should be pathologically confirmed as nasopharyngeal carcinoma and the expression of EGFR/p-EGFR should be detected via immunohistochemistry; the study should analyze the prognostic significance of EGFR/p-EGFR in nasopharyngeal carcinoma; hazard ratio (HR) and 95% confidence interval (CI) should be reported in the study or could be derived from survival curves; and the outcomes of the study should include overall survival (OS), disease-free survival (DFS), progression-free survival (PFS), and distant metastasis-free survival (DMFS).

**Results:**

A total of 18 studies evaluating 1451 samples were included. For studies that reported OS as an outcome, EGFR overexpression indicated worse OS of nasopharyngeal carcinoma patients. The heterogeneity between studies was high (*I*^2^ = 91%, *P* < .01), and a random-effect model was used to combine the effect size (HR = 1.71, 95% CI [1.21, 2.41], *P* < .01). Further sensitivity analysis and prespecified subgroup analysis were performed to detect the source of heterogeneity, and the results showed that the heterogeneity could not be eliminated. Publication bias assessed by funnel plots and Begg test and Egger test was low (Begg test: *P* = .846 and Egger test: *P* = .074). p-EGFR was not correlated with the OS of nasopharyngeal carcinoma patients (HR = 1.01, 95% CI [0.88, 1.15], *P* = .92). For studies that reported DFS, EGFR overexpression was associated with worse DFS in patients with nasopharyngeal carcinoma (HR = 2.53, 95% CI [1.84, 3.47], *P* < .01). For studies that reported PFS, EGFR overexpression was not correlated with the PFS of nasopharyngeal carcinoma patients (HR = 1.86, 95% CI [0.90, 3.82], *P* = .09). For studies that reported DMFS, EGFR overexpression was not correlated with the DMFS of nasopharyngeal carcinoma patients, and high heterogeneity between studies was detected (*I*^*2*^ = 97%, *P* < .01). A random-effect model was used to combine the effect size (HR = 1.80, 95% CI [0.56, 5.76], *P* = .32). A sensitivity analysis was conducted. Publication bias was detected to be low (Begg test: *P* = .817 and Egger test: *P* = .954). There was no correlation between p-EGFR overexpression and DMFS in patients with nasopharyngeal carcinoma (HR = 1.20, 95% CI [0.95, 1.52], *P* = .12).

**Conclusion:**

In nasopharyngeal carcinoma patients, EGFR overexpression could be used as a biomarker that predicts poor OS and DFS, but not a prognostic biomarker for PFS and DMFS. The overexpression of p-EGFR was not shown to be associated with the prognosis of nasopharyngeal carcinoma patients and could not be used as a prognostic biomarker.

**Ethics and dissemination:**

This study was registered on the International Platform of Registered Systematic Review and Meta-analysis Protocols (INPLASY), and reported as stated by the Preferred Reporting Items for Systematic reviews and Meta-Analyses. Neither ethical approval nor informed consent was required since this study was conducted based on previous publications.

**INPLASY registration number:**

INPLASY 202150010

## 1 Introduction

Nasopharyngeal carcinoma is a malignant nasopharyngeal epithelial tumor that is strongly invasive and prone to metastases.^[1,2]^ Worldwide, around 133,000 people were diagnosed with nasopharyngeal carcinoma and 80,000 people succumbed to death in 2020.^[3]^ Due to its hidden anatomic site and unobvious early symptoms (such as nose hemorrhage and headaches), most patients were de-novo advanced nasopharyngeal carcinoma at the time of diagnosis and had unfavorable prognosis.^[4]^ Multidisciplinary treatment modalities incorporating radiotherapy, chemotherapy, and targeted therapy have been the main treatment pattern of nasopharyngeal carcinoma,^[5,6]^ while there are still severe challenges in the current treatment and management. The TNM staging system mainly predicts the prognosis of patients with nasopharyngeal carcinoma based on three aspects: tumor size, lymph node metastasis, and distant metastasis. However, the development of nasopharyngeal carcinoma is an interactive process involving multiple biological behaviors, and the TNM staging system alone cannot perfectly predict the disease prognosis.^[7]^ Therefore, identifying accurate prognostic biomarkers is of great significance for clinical diagnosis and treatment.^[8]^ Epidermal growth factor receptor (EGFR) is a tyrosine kinase and a transmembrane receptor, and its overexpression may imply tumor progression.^[9]^ A study found that EGFR was overexpressed in most nasopharyngeal carcinoma cell lines and patients.^[10]^ It was reported that specific mutations of EGFR can induce continuous phosphorylation of EGFR, while an increased level of phosphorylated EGFR activates downstream signals and can subsequently induce carcinoma.^[11,12]^ When growth factors bind to EGFR, EGFR will be auto-phosphorylated by tyrosine kinases. Phosphorylated EGFR (p-EGFR) activates cell signaling pathways and has implications in cell cycle, apoptosis, angiogenesis, and cell proliferation.^[11,12]^ It has been shown that EGFR and p-EGFR are esstential in the carcinogenesis and development of nasopharyngeal carcinoma. Currently, many drugs against EGFR tyrosine kinase have been developed and put into clinical use. Previous studies showed that the efficacy of Gefitinib and Cetuximab that targets EGFR in nasopharyngeal carcinoma was unsatisfactory,^[13–15]^ while the combination of Cetuximab and radiotherapy or chemotherapy was proved feasible for locally advanced nasopharyngeal carcinoma.^[16]^ In addition, the combination of Nimotuzumab and neoadjuvant therapy for patients with locally advanced nasopharyngeal carcinoma yielded satisfactory efficacy and decreased adverse events.^[17]^ In summary, there have been controversies over the efficacy of anti-EGFR targeted therapy for nasopharyngeal carcinoma, which suggests that further research and discussions are warranted on the correlation between EGFR and the prognosis of nasopharyngeal carcinoma.

There have been several studies focusing on the correlation between EGFR expression and the prognosis of nasopharyngeal carcinoma, while conflicting results were reported in these studies.^[18,19]^ Whether EGFR overexpression is associated with the prognosis of nasopharyngeal carcinoma remains controversial. Meanwhile, the correlation of p-EGFR (the activated form of EGFR) overexpression with the prognosis of nasopharyngeal carcinoma has also been undetermined. Previous meta-analyses^[20–22]^ showed that EGFR overexpression predicted unfavorable overall survival (OS) and disease-free survival (DFS) but did not correlate with the absence of distant metastasis in patients with nasopharyngeal carcinoma. However, these three studies were published more than a few years ago and need to be updated. Additionally, none of the three studies has performed a systematic review or meta-analysis into the prognostic effect of p-EGFR expression toward nasopharyngeal carcinoma. Here, a systematic review and meta-analysis was designed to probe into the correlation between the prognosis of nasopharyngeal carcinoma and the expression of EGFR/p-EGFR, in an attempt to identify the prognostic effect of EGFR/p-EGFR level in nasopharyngeal carcinoma.

## 2 Methodologies

This study was registered on the International Platform of Registered Systematic Review and Meta-analysis Protocols (INPLASY) (No. INPLASY202150010), and reported as stated by the Preferred Reporting Items for Systematic reviews and Meta-Analyses.^[23]^ Neither ethical approval nor informed consent was required since this study was conducted based on previous publications.

### 2.1 Search strategy

A systematic search of the following six electronic databases was performed for prospective and retrospective studies on the correlation of EGFR/p-EGFR and prognosis of nasopharyngeal carcinoma from inception to April 2021: PubMed, Embase, China National Knowledge Infrastructure, Database of Chinese sci-tech periodicals, WanFang Database and China Biology Medicine disc. The following MeSH terms were used: epidermal growth factor receptor, epidermal growth factor receptor related protein, EGFR protein, ErbB Receptors, EGFR, phosphorylated signal epidermal growth factor receptor, phosphorylated EGFR, p-EGFR, Nasopharyngeal Carcinoma, Nasopharyngeal Neoplasms, Nasopharyngeal Cancer, NPC. The language of literature was restricted to Chinese and English. An additional manual retrieval of the references to the included studies was also performed to ensure that all eligible literature could be included. The search strategy is detailed in Table 1 (take PubMed as an example).

**Table 1:** The search strategy for PubMed.

Table 1	
PubMed Search Strategy	
Number	Search terms
#1	((((((((epidermal growth factor receptor[MeSH Terms])) OR (epidermal growth factor receptor related protein, human[MeSH Terms])) OR (EGFR protein, human[MeSH Terms])) OR (ErbB Receptors[MeSH Terms])) OR (EGFR[Title])) OR (phosphoralated EGFR[Title])) OR (phosphoralated signal epidermal growth factor receptor[Title])) OR (p-EGFR[Title]))
#2	((((Nasopharyngeal Carcinoma[MeSH Terms])) OR (Nasopharyngeal Neoplasms[MeSH Terms])) OR (Nasopharyngeal Cancer[MeSH Terms])) OR (NPC[Title])
#3	#1and #2

### 2.2 Inclusion and exclusion criteria

Inclusion criteria: (1) All the tissue samples investigated should be removed from patients who were pathologically diagnosed with nasopharyngeal carcinoma. (2) Studies should use immunohistochemistry (IHC) to assess EGFR/p-EGFR expression in nasopharyngeal carcinoma tissue samples and analyze their associations with patient prognosis. (3) The hazard ratio (HR) and 95% confidence interval (CI) for one or more of the four outcomes (OS, DFS, progression-free survival [PFS] and distant metastasis-free survival [DMFS]) should be reported in the study or could be derived from survival curves. Exclusion criteria: (1) None of the above four outcomes is reported in the study. (2) Duplicate publications or studies with incomplete data. (3) Animal or cell experiments, editorials, comments, case reports, conference abstracts, and reviews. (4) HR and 95% CI could not be derived.

### 2.3 Data extraction

Studies retrieved by the above search strategy was managed using Endnote software. After excluding duplicate studies, two researchers in our team preliminarily screened all the records according to the above criteria and excluded ineligible studies by reading the titles and abstracts, followed by reading the full text. We tried to contact the author of the original study for complete information where incomplete data existed. Study selection was performed by the two researchers independently, and a third party would be consulted if there were disputes or disagreements. Data extraction was completed by the two researchers independently according to the standard data extraction sheet designed by our team and recorded the extracted data onto a Microsoft Excel sheet. Extracted information included author (year), country, ethnicity, study design, sample size, clinical stage, histological differentiation, cut-off value, testing modality, etc. The main outcomes were OS, DFS, PFS, and DMFS. The HR along with 95% CI of univariate or multivariate analyses was extracted when directly given, and was otherwise calculated by the computational formula according to Tierney et al^[24]^ or HR calculations spreadsheet using Engauge Digitizer 12.1 software after having extracted the survival rate of various follow-up period from survival curves.

### 2.4 Quality assessment

All parts of the included studies were assessed according to the Preferred Reporting Items for Systematic reviews and Meta-analyses checklist, and quality assessment was performed according to the evaluation tool of prognosis studies^[25]^ (proposed by Jill A. Hayden, Pierre Côté, Claire Bombardier). The evaluation of prognostic studies used an item list that addressed 6 sources of potential bias, including: (1) selection of the study participants; (2) study attrition during the follow-up (loss to follow-up or withdrawal); (3) clear definition of the prognostic factors and their evaluating approaches; (4) measurement of outcomes; (5) measurement and explanation of all confounding factors; (6) data analysis and selective report of the results, etc. The risk of bias was assessed in three grades: Yes, Partly, and No/Unsure. The point assignment was: 2 points for Yes, 1 point for Partly, and 0 point for No/Unsure. The total score ranged from 0-12 points, and 8-12 points suggested a high-quality study while 0-7 points indicated a low-quality one.

### 2.5 Statistical analysis

R software 4.0.5 was used to analyze the data. The HR with 95% CI value for the main outcomes would be combined. *Q* test and *I*^*2*^ test were performed to assess the heterogeneity of study results. A random-effect model would be applied in case of significant heterogeneity (*I*^2^ > 50%, *P* < .1), otherwise a fixed-effect model would be applied upon low heterogeneity (*I*^2^ < 50%, *P* > .1). Further sensititive analysis and subgroup analysis were designed in case of the presence of heterogeneity. A funnel plot would be drawn for assessing publication bias when there were more than 10 studies, otherwise descriptive analysis would be implemented.

## 3 Results

### 3.1 Search results

**Figure 1:**
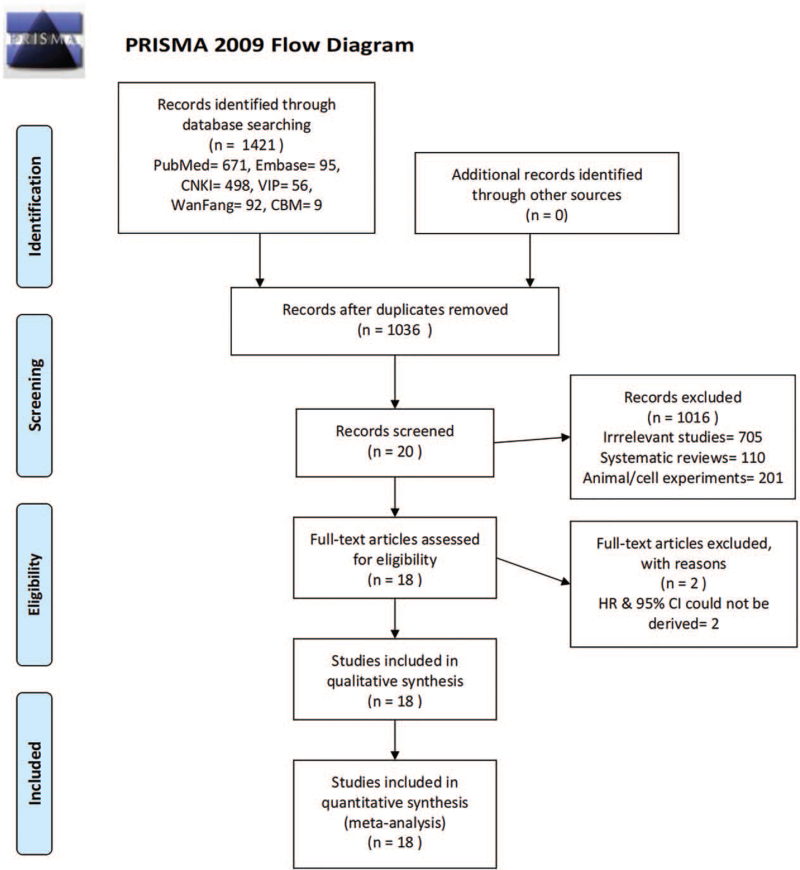
Flow diagram.

In all, 1647 relevant studies were retrieved from PubMed, Embase, China National Knowledge Infrastructure Database, Database of Chinese sci-tech periodicals, WanFang Database and China Biology Medicine disc using the above-mentioned search strategy, and 584 duplicated records were excluded using Endnote software X9.3. Two researchers excluded 941 studies following reading the titles and abstracts, and eventually enrolled 18 studies after reading the full text. The workflow is depicted in Figure 1.

### 3.2 Study characteristics

Study characteristics of the final 18 studies are shown in Table 2. Of the 18 studies, 5 were published in Chinese and 13 were published in English. Swedish, Singaporean, and Japanese researchers each contributed 1 publication, 2 studies were from Korea, and 13 were from China. There were 17 studies that enrolled Asian patients and only 1 study enrolled Caucasians. The sample size ranged from 30 to 176. All the tissue samples were tested with IHC, and the cut-off value of EGFR/p-EGFR expression ranged 5% to 50%. Among all eligible studies, 17 studies reported the correlation between EGFR expression and the prognosis of nasopharyngeal carcinoma, and there were 15, 8, 2, and 5 studies that reported OS, DFS, PFS, and DMFS as the outcome, respectively. Three studies reported the correlation between the expression of p-EGFR and the prognosis of nasopharyngeal carcinoma, and there are 3, 1 and 2 studies reported OS, PFS, and DMFS as the study outcome, respectively. Among all the included studies, there were 16 high-quality and 2 low-quality studies. Quality assessment of these prognostic studies are shown in Table 3.

**Table 2:** Relevant information of the included literature.

Author (year)	Country	Ethnicity	Study design	No. of patients	Clinical stage (I-II/III-IV)	Histologic classification (U/D)	Marker	Detection	Outcome
Fujii M^[26]^ 2002	Japan	Asian	Retro	53	24/29	8/45	EGFR	IHC	DFS
Ma BB.Y.^[27]^ 2003	China	Asian	Pro	78	29/49	78/0	EGFR	IHC	OS,DFE
Chua DT^[28]^ 2004	China	Asian	Retro	54	0/54	54/0	EGFR	IHC	DFS,DMFS
Leong JL^[29]^ 2004	Singapore	Asian	Pro	75	26/48	75/0	EGFR	IHC	OS,DFS
Wang SS^[30]^ 2006	China	Asian	Retro	55	7/48	NA	EGFR	IHC	OS
Fang FM^[31]^ 2007	China	Asian	Retro	30	11/19	17/13	EGFR	IHC	OS,DFS
Yuan TZ^[32]^ 2008	China	Asian	Retro	110	27/83	110/0	EGFR p-EGFR	IHC	OS,DMFS
Yuan YL^[33]^ 2008	China	Asian	Retro	75	24/51	51/24	EGFR	IHC	OS
Zahra TK^[34]^ 2009	Sweden	Caucasian	Retro	45	12/33	34/11	EGFR	IHC	OS,DFS,DMFS
Huang TL^[35]^ 2010	China	Asian	Retro	176	21/155	93/83	EGFR p-EGFR	IHC	OS,DMFS
Kim YJ^[36]^ 2010	Korea	Asian	Retro	38	6/32	31/7	EGFR	IHC	OS,PFS
Qi LB^[37]^ 2010	China	Asian	Retro	55	13/42	55/0	EGFR	IHC	OS
Cao XJ^[38]^ 2012	China	Asian	Retro	127	0/127	NA	EGFR	IHC	OS,DFS
Pan JJ^[39]^ 2012	China	Asian	Retro	111	30/81	5/106	EGFR	IHC	OS,DFS,DMFS
Wu W^[40]^ 2015	China	Asian	Retro	107	12/95	107/0	p-EGFR	IHC	OS,PFS
Zhang P^[41]^ 2015	China	Asian	Retro	96	21/75	NA	EGFR	IHC	OS
Kang H^[18]^ 2016	Korea	Asian	Retro	46	20/26	18/28	EGFR	IHC	OS
Wang Y^[19]^ 2019	China	Asian	Retro	120	18/102	104/16	EGFR	IHC	OS,PFS

**Table 3:** Results of quality assessment of prognostic studies.

Author (year)	Study participation	Study attrition	Prognostic factor measurement	Outcome measurement	Confounding measurement and account	Analysis	Total
Fujii M 2002	2	1	2	1	1	2	9
Ma BB.Y.2003	2	1	2	1	1	2	9
Chua DT 2004	2	1	2	1	1	2	9
Leong JL 2004	2	1	2	1	1	1	8
Wang SS 2006	2	1	1	1	1	1	7
Fang FM 2007	2	1	2	1	1	1	8
Yuan TZ 2008	2	2	1	1	1	1	7
Yuan YL 2008	2	1	2	1	1	1	8
Zahra TK 2009	2	2	2	1	1	1	9
Huang TL 2010	2	1	2	2	1	2	10
Kim YJ 2010	2	1	2	2	1	2	10
Qi LB 2010	2	1	2	1	1	1	8
Cao XJ 2012	2	1	2	1	1	1	8
Pan JJ 2012	2	2	2	1	1	1	9
Wu W 2015	2	2	2	1	1	1	9
Zhang P 2015	2	2	2	1	1	1	9
Kang H 2016	2	1	2	2	1	2	10
Wang Y 2019	2	1	2	1	1	1	8

### 3.3 Meta-analysis results

#### 3.3.1 EGFR/p-EGFR and OS

**Figure 2:**
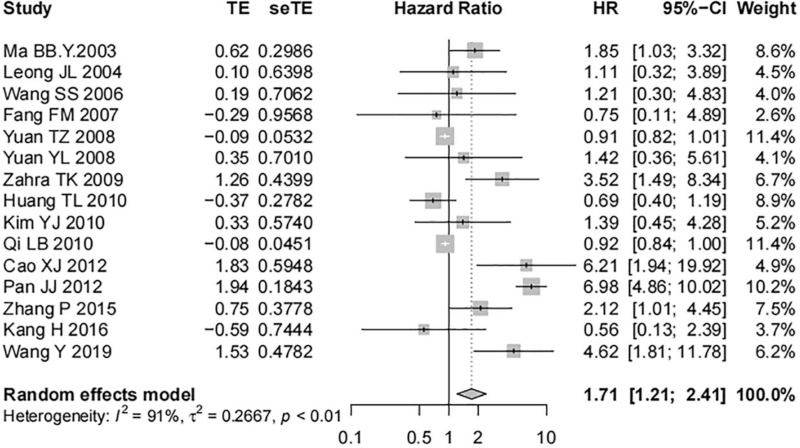
Forest map on the correlation of EGFR expression with OS of nasopharyngeal carcinoma patients.

**Figure 3:**
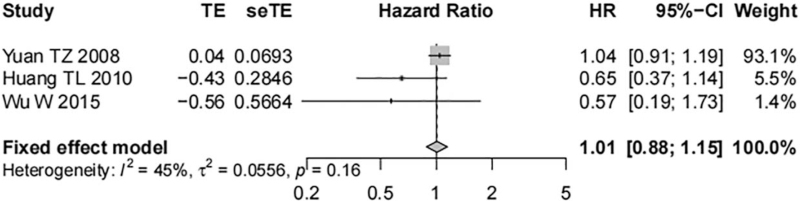
Forest map on the correlation of p-EGFR expression with OS of nasopharyngeal carcinoma patients.

Fifteen studies including 1237 tissue samples removed from nasopharyngeal carcinoma patients reported the correlation of EGFR expression with the OS of nasopharyngeal carcinoma patients. High heterogeneity was shown between studies (*I*^*2*^ = 91%, *P* < .01), and a random-effect model was adopted. Meta-analysis identified a significant correlation of EGFR overexpression with worse OS of patients and a statistically significant difference was shown (HR = 1.71, 95% CI [1.21, 2.41], *P* < .01, Fig. 2). Three studies investigating 393 nasopharyngeal carcinoma tissue samples reported the association of the expression of p-EGFR and the OS of nasopharyngeal carcinoma patients. The results showed low heterogeneity among the studies (*I*^*2*^ = 45%, *P* = .16), and a fixed-effect model was used. No correlation was shown between p-EGFR overexpression and the OS of patients with nasopharyngeal carcinoma and no statistically significant difference was shown (HR = 1.01, 95% CI [0.88, 1.15], *P* = .92, Fig. 3).

#### 3.3.2 EGFR and DFS

**Figure 4:**
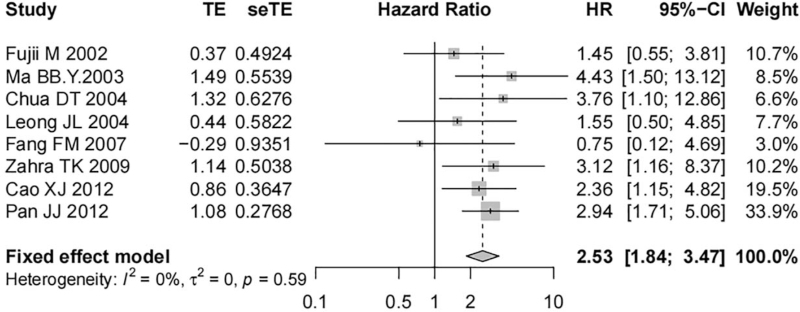
Forest map on the correlation of EGFR expression with DFS of nasopharyngeal carcinoma patients.

Eight studies including 683 nasopharyngeal carcinoma tissue samples reported the correlation of EGFR expression with the DFS of patients. No significant heterogeneity was detected among the studies (*I*^*2*^ = 0%, *P* = .59), and a fixed-effect model was used. The results indicated a statistically significant difference, and EGFR overexpression was associated with worse DFS in patients with nasopharyngeal carcinoma (HR = 2.53, 95% CI [1.84, 3.47], *P* < .01, Fig. 4).

#### 3.3.3 EGFR/p-EGFR and PFS

**Figure 5:**
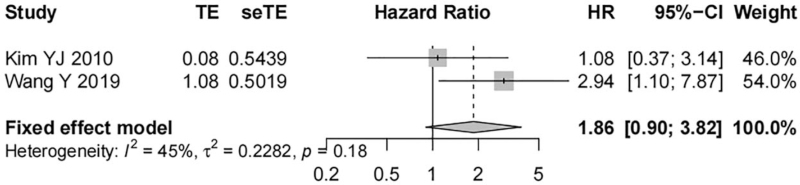
Forest map on the correlation of EGFR expression with PFS of nasopharyngeal carcinoma patients.

Two studies including 158 tissue samples removed from patients with nasopharyngeal carcinoma reported the correlation between EGFR expression and the PFS of patients. Heterogeneity between the studies was low (*I*^*2*^ = 45%, *P* = .18), and a fixed-effect model was taken. Meta-analysis demonstrated that the PFS of nasopharyngeal carcinoma patients was independent of EGFR overexpression and there was no statistically significant difference (HR = 1.86, 95% CI [0.90, 3.82], *P* = .09, Fig. 5). Since only one study including 107 nasopharyngeal carcinoma tissue samples investigated the association of p-EGFR and PFS in patients with nasopharyngeal carcinoma, the meta-analysis was not performed.

#### 3.3.4 EGFR/p-EGFR and DMFS

**Figure 6:**
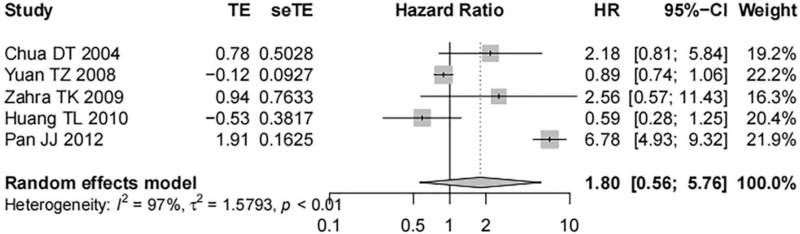
Forest map on the correlation of EGFR expression with DMFS of nasopharyngeal carcinoma patients.

**Figure 7:**
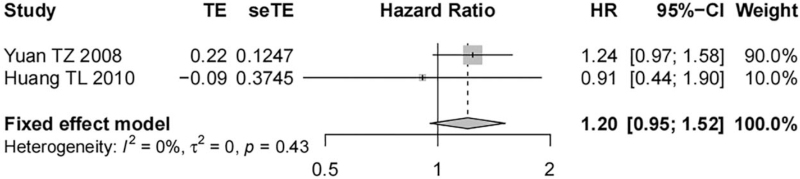
Forest map on the correlation of p-EGFR expression with DMFS of nasopharyngeal carcinoma patients.

Five studies containing 496 nasopharyngeal carcinoma tissue samples reported the correlation between EGFR expression and the DMFS of patients. Significant heterogeneity was shown among studies (*I*^*2*^ = 97%, *P* < .01), and a random-effect model was used. EGFR overexpression was not associated with the DMFS of nasopharyngeal carcinoma patients and no statistically significant difference was demonstrated (HR = 1.80, 95% CI [0.56, 5.76], *P* = .32, Fig. 6). Two studies containing 286 nasopharyngeal carcinoma tissue samples reported the correlation of p-EGFR expression with the DMFS of patients. There was no evident heterogeneity among the studies (*I*^*2*^ = 0%, *P* = .43), and a fixed-effect model was used. No statistically significant difference was noted. It showed that the DMFS of patients suffering from nasopharyngeal carcinoma was independent of p-EGFR overexpression (HR = 1.20, 95% CI [0.95, 1.52], *P* = .12, Fig. 7).

### 3.4 Sensitivity and subgroup analysess

Since significant heterogeneity was detected in the meta-analysis of the studies on the association of EGFR expression with OS and DMFS in nasopharyngeal carcinoma patients, we performed sensitivity analysis to see if the exclusion of any study would impact the synthesized results of HR and 95% CI. We used R software to perform sensitivity analyses, and the final result was not changed after excluding studies that may affect pooled results, suggesting the robustness of our meta-analyses. We further performed subgroup analyses of studies with sufficient data in terms of the correlation of EGFR with OS. Studies were classified into different subgroups based on ethnicity (Asian or Caucasian), sample size (>100 or < = 100), the cut-off value of IHC (10% or 25%), and histological differentiation. The result of subgroup analysis is demonstrated in Table 4.

**Table 4:** Subgroup analysis for the expression of EGFR expression with OS of nasopharyngeal carcinoma patients.

Subgroup		Number	Hazard ratio	95% Confidence interval	*P*	*I*^2^(%)
Ethnicity	Asian	14	1.63	(1.14, 2.31)	<.01	91
Sample	>100	5	2.52	(0.84, 7.51)	.10	97
	≤100	10	1.40	(0.95, 2.07)	.02	56
Cutoff	10%	4	2.16	(0.74, 6.30)	.16	87
	25%	4	1.59	(0.59, 4.28)	.36	74
Histological	Undifferentiated	11	1.26	(0.98, 1.61)	.07	74
	Differentiated	2	2.18	(0.18, 25.84)	.54	91

### 3.5 Publication bias

**Figure 8:**
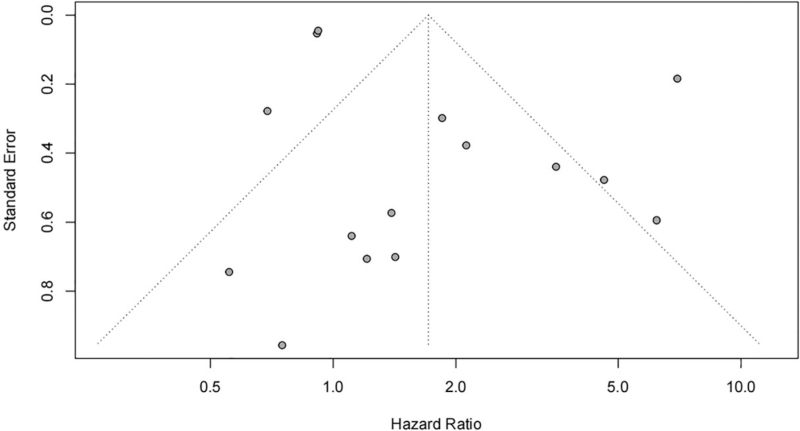
Funnel plot of the studies investigating the correlation of EGFR with OS of nasopharyngeal carcinoma patients.

Publication bias is common in meta-analysis. A funnel plot for the studies investigating the correlation of EGFR with OS was drawn using R software, and publication bias was assessed through Begg test and Egger test. It demonstrated that the 15 studies distributed symmetrically, and the *P*-values in Begg and Egger tests were separately .846 and .074, which suggested no publication bias (Fig. 8). The publication bias of studies investigating the correlation of EGFR with DMFS was also evaluated. The *P*-values in Begg and Egger tests were separately .817 and .954, which also suggested that no publication bias existed.

## 4 Discussion

This systematic review and meta-analysis showed that EGFR overexpression predicted worse OS and DFS of patients with nasopharyngeal carcinoma, but was not correlated with the PFS or DMFS. For p-EGFR expression, this study showed that p-EGFR overexpression was not significantly associated with OS or DMFS of nasopharyngeal carcinoma patients. Due to a lack of studies to synthesize HR and 95% CI, the association of p-EGFR expression with PFS and DFS was not evaluated in this study.

The present study results were in agreement with the three existing systematic review and meta-analyses and suggested that overexpressed EGFR could act as a biomarker of unfavorable OS and DFS in nasopharyngeal carcinoma patients. However, high heterogeneity was detected when combining studies that reported OS as an outcome, and the source of heterogeneity was thus identified in subsequent sensitivity and subgroup analyses. After excluding studies that may introduce heterogeneity in the sensitivity analysis,^[39]^ the heterogeneity remained significant, and a further subgroup analysis was needed. The subgroup analyses were performed in terms of ethnicity, sample size, cut-off value, and the histological differentiation of tumor. In this systematic review and meta-analysis, 17 studies were from China, Korea, Japan, and Singapore, which were all conducted among Asian patients, while only one study was from Swedish and enrolled Caucasians.^[34]^ Since the incidence of nasopharyngeal carcinoma is strongly associated with ethnicity and districts,^[42]^ we conducted subgroup analysis according to the ethnicity of included patients. After excluding the study conducted in Caucasians, the heterogeneity between studies remained high (*I*^*2*^ = 91%), which suggested that the heterogeneity did not come from the ethnicity of study participants. We subsequently analyzed the impact of sample size on the study results, and the subgroup analysis according to sample size with a cut-off of 100 participants showed that the heterogeneity of either group was higher than 50%. This indicated that the discrepancy in sample size was not the source of heterogeneity. We also classified studies into subgroups according to the proportion of different histological differentiation, and subgroup analysis showed that significant heterogeneity still existed, suggesting that histological differentiation was not the source of heterogeneity. We finally stratified included studies according to the IHC. Although all included studies used IHC to evaluate the expression of EGFR, the cut-off for determining high and low expression of EGFR was highly inconsistent. In addition, a study proved that IHC was preferred among all the testing methods of EGFR,^[43]^ while there was also evidence showing that the result of IHC could be affected by various factors when evaluating EGFR expression.^[44]^ Therefore, we stratified studies according to the cut-off value of 10% or 25%, and subgroup analysis also showed that heterogeneity did not came from the difference of the cut-off value. In a word, the source of heterogeneity could not be determined in our sensitivity analysis and subgroup analyses, which indicated that the heterogeneity could not be eliminated.

This study showed that high EGFR expression was not strongly correlated with the PFS of patients with nasopharyngeal carcinoma. However, only 2 studies reported PFS as an outcome in our study. One study^[36]^ showed no correlation between high EGFR expression and PFS in nasopharyngeal carcinoma patients, while the other^[19]^ showed that high EGFR expression predicted worse PFS. Since only a few studies reported PFS as an outcome, this pooled result was not robust enough. At present, different studies have reached a consensus that distant metastasis that leads to disease recurrence poses the most severe challenge to the management of nasopharyngeal carcinoma, resulting in unfavorable prognosis.^[45,46]^ In this study, there were 5 studies that reported DMFS as the study outcome, and pooled results demonstrated that EGFR overexpression was independent of the DMFS in nasopharyngeal carcinoma patients. Studies found that the distant metastasis of nasopharyngeal carcinoma was closely associated with many factors such as the clinical stage of patients, lymphadenopathy grading, and the histological differentiation of tumor.^[47,48]^ Here, in the 5 studies that reported DMFS as an outcome, around 82% (406/496) samples were in stage III-IV, and most samples were differentiated tumors. These may be the confounding factors when analyzing the prognostic effect of EGFR expression on the DMFS of nasopharyngeal carcinoma patients.

Regarding the association of p-EGFR expression with the prognosis of nasopharyngeal carcinoma patients, the pooled 95% CI crossed 1 for the outcome of OS and DMFS, suggesting no association of p-EGFR overexpression with OS or DMFS. In this systematic review and meta-analysis, only 3 studies reported the prognostic effect of p-EGFR on nasopharyngeal carcinoma, among which 3, 1, and 2 studies reported OS, PFS, and DMFS as an outcome, respectively. The sample size was small, which might lead to unrobust and unreliable pooled results to some degree. Further validations and analyses based on a large sample size are warranted in the future.

This systematic review and meta-analysis used an evaluation tool of prognostic studies to complete quality assessment of included studies. This tool consisted of 6 sources of potential bias and took confounding factors into consideration, which was superior to the Newcastle-Ottawa Scale.^[49]^ For the pooled analyses of EGFR expression and prognosis (OS, DMFS) with significant heterogeneity, Begg test and Egger test were applied to determine publication bias, and a funnel plot was drawn when there were more than 10 studies. Both test results and funnel plots showed no publication bias, which was consistent with previous systematic reviews.

This systematic review and meta-analysis also had some limitations. (1) Due to the language restrictions, only publications in Chinese and English were included in this study. Most studies enrolled Asian patients, while Caucasians were only investigated in one study. This led to the insufficient evaluation of patients other than Asians. (2) The studies included in this meta-analysis assessed patients in stage I-IV and both differentiated/undifferentiated samples, while the EGFR/p-EGFR expression according to different subgroups was unknown. The potential impact of these confounding factors on the study results could not be eliminated. (3) Although all the included studies used IHC to detect EGFR/p-EGFR expression, different fixatives and cut-off values for positivity might introduce heterogeneity. (4) Since some studies only provided Kaplan-Meier survival curves and did not show HR and 95% CI directly, the HR and 95% CI were calculated through software, which might be slightly inconsistent with the real data.

## 5 Conclusion

Overexpressed EGFR could be used as a prognostic biomarker for unfavorable OS and DFS but not DMFS in patients with nasopharyngeal carcinoma. The overexpression of p-EGFR was not shown to have implications in the prognosis of nasopharyngeal carcinoma patients, thus it could not be used as a biomarker for the prognosis.

## Author contributions

XHH, GYW, GLX, TBW, CMM, ZR and WS conceived and designed the protocol, and XHH drafted the protocol manuscript. XHH developed the search strategy, with input from GYW and GLX. XHH, CMM, and TBW planned the data extraction. XHH, ZR and WS planned the quality appraisal of all included studies. XHH, GYW, GLX, TBW, CMM, ZR and WS, critically revised the manuscript for methodological and intellectual content. All authors approved the final version.

**Conceptualization:** Xiaohua Hong, Guangyao Wang, Zhen Rong, Wei Shi.

**Data curation:** Wei Shi, Tongbiao Wang.

**Formal analysis:** Xiaohua Hong, Guangyao Wang.

**Project administration:** Xiaohua Hong, Guangyao Wang, Guanglan Xu.

**Supervision:** Xiaohua Hong, Zhen Rong, Chunmei Mo.

**Writing – original draft:** Xiaohua Hong.

**Writing – review & editing:** Chunmei Mo, Zhen Rong.
